# Development of modified HCH-1 kinetic model for long-term enzymatic cellulose hydrolysis and comparison with literature models

**DOI:** 10.1186/s13068-019-1371-5

**Published:** 2019-02-18

**Authors:** Chao Liang, Chao Gu, Jonathan Raftery, M. Nazmul Karim, Mark Holtzapple

**Affiliations:** 1 Department of Chemical Engineering, Texas A&M University, College Station, TX 77843-3122 USA; 2 Department of Educational Psychology, Texas A&M University, College Station, TX 77843-3122 USA

**Keywords:** Enzymatic cellulose hydrolysis, Simulation, HCH-1, Model comparison, Sensitivity analysis

## Abstract

**Background:**

Enzymatic hydrolysis is a major step for cellulosic ethanol production. A thorough understanding of enzymatic hydrolysis is necessary to help design optimal conditions and economical systems. The original HCH-1 (Holtzapple–Caram–Humphrey–1) model is a generalized mechanistic model for enzymatic cellulose hydrolysis, but was previously applied only to the initial rates. In this study, the original HCH-1 model was modified to describe integrated enzymatic cellulose hydrolysis. The relationships between parameters in the HCH-1 model and substrate conversion were investigated. Literature models for long-term (> 48 h) enzymatic hydrolysis were summarized and compared to the modified HCH-1 model.

**Results:**

A modified HCH-1 model was developed for long-term (> 48 h) enzymatic cellulose hydrolysis. This modified HCH-1 model includes the following additional considerations: (1) relationships between coefficients and substrate conversion, and (2) enzyme stability. Parameter estimation was performed with 10-day experimental data using α-cellulose as substrate. The developed model satisfactorily describes integrated cellulose hydrolysis data taken with various reaction conditions (initial substrate concentration, initial product concentration, enzyme loading, time). Mechanistic (and semi-mechanistic) literature models for long-term enzymatic hydrolysis were compared with the modified HCH-1 model and evaluated by the corrected version of the Akaike information criterion. Comparison results show that the modified HCH-1 model provides the best fit for enzymatic cellulose hydrolysis.

**Conclusions:**

The HCH-1 model was modified to extend its application to integrated enzymatic hydrolysis; it performed well when predicting 10-day cellulose hydrolysis at various experimental conditions. Comparison with the literature models showed that the modified HCH-1 model provided the best fit.

**Electronic supplementary material:**

The online version of this article (10.1186/s13068-019-1371-5) contains supplementary material, which is available to authorized users.

## Background

Biomass is the only renewable energy resource that can be directly converted to liquid fuels and chemicals. The largest biomass feedstock is lignocellulose, which is found globally in many forms. Converting lignocellulose into biofuels could relieve shortages of liquid fuels and reduce dependence on fossil energy.

In the United States, ethanol is the dominant biofuel, which is usually produced from corn, an important food for animals and humans. To prevent food shortages, cellulosic ethanol is an attractive alternative. In general, there are four major steps for cellulosic ethanol production: pretreatment, hydrolysis, fermentation, and separation. Among these processes, hydrolysis accounts for a large portion (~ 30%) of the total costs [1]. To compete with corn ethanol and petroleum-derived gasoline, enzymatic hydrolysis needs optimization and cost reduction [2]; therefore, a thorough understanding of enzymatic hydrolysis is necessary to help design optimal conditions and economical systems.

During the last several decades, various theoretical and empirical models have been developed to simulate enzymatic hydrolysis of cellulose [3–6]. Because they lack a theoretical foundation, empirical models cannot be applied beyond the range of the data; therefore, this paper only focuses on mechanistic (and semi-mechanistic) models.

**Fig. 1:**
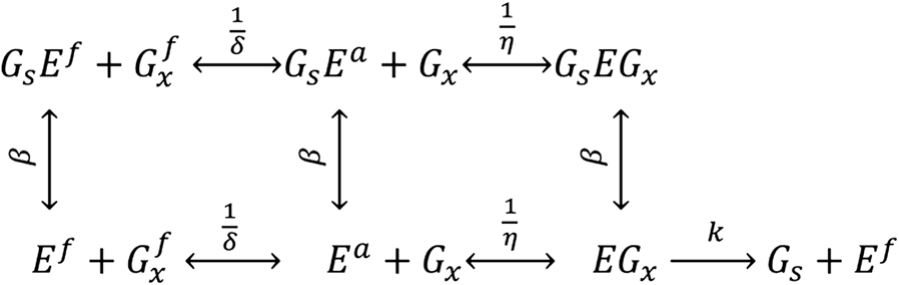
Reaction mechanism for the HCH-I model [3]

In 1984, Holtzapple et al. [3] proposed a generalized mechanistic model for cellulose hydrolysis termed the HCH-1 (Holtzapple–Caram–Humphrey-1) model. Figure 1 shows the reaction mechanism of the HCH-1 model [3]. As shown in the figure, free enzyme (*E*^*f*^) adsorbs onto a free cellulose site ($G_{x}^{f}$) to become adsorbed enzyme (*E*^*a*^), and then complexes with the cellulose to become enzyme–substrate complex (*EG*_*x*_). This complex catalyzes the hydrolysis of the cellulose site to obtain soluble product (*G*_*s*_) with reaction rate *k*. All enzyme species can complex with product to become inhibited enzyme ($G_{s} E^{f} ,\;\; G_{s} E^{a}$, and *G*_*s*_*EG*_*x*_). For simplicity, the product-binding constant (*β*) is assumed to be the same for all the enzyme species. In addition, the adsorption constant (*δ*) and the complexing constant (*η*) are assumed not to be affected by the binding of product to the enzyme [3].

The rate-limiting step is the hydrolysis; therefore, the reaction velocity (*V*) is proportional to the concentration of uninhibited enzyme–substrate complex (*EG*_*x*_). To express the reaction velocity in terms of known variables, substitutions can be made for *EG*_*x*_ using material balances of substrate and enzyme species, thus, yielding the HCH-1 model (Eq. 1). The detailed model development is described in [3].

$$V = \frac{{\kappa \left[ {G_{x} } \right]\left[ E \right]i}}{{\alpha + \varphi \left[ {G_{x} } \right] + \varepsilon \left[ E \right]}}$$

$$i = \frac{1}{{\left. {1 + \beta_{1} [G_{1} } \right] + \beta_{2} \left[ {G_{2} } \right]}}$$

1 $$\begin{array}{*{20}c} { \varphi = \frac{{\left[ {G_{x} } \right] - \alpha - \varepsilon \left[ E \right] + \sqrt {\left( {\left[ {G_{x} } \right] - \alpha - \varepsilon \left[ E \right]} \right)^{2} + 4\alpha \left[ {G_{x} } \right]} }}{{2\left[ {G_{x} } \right]}}} \\ \end{array}$$

where *G*_*x*_ is the cellulose concentration (g/L, equivalent to glucose), *G*_1_ is the glucose concentration (g/L), *G*_2_ is the cellobiose concentration (g/L, equivalent to glucose), *E* is the enzyme concentration (g/L), *α* is the lumped adsorption constant ($\alpha = \frac{\eta \delta }{\eta + 1}$, g/L), *κ* is the lumped kinetic constant ($\kappa = \frac{k}{\eta + 1}$, h^−1^), *β*_1_ is the glucose binding constant (L/g), *β*_2_ is the cellobiose binding constant (L/g), *ɛ* is the number of cellulose sites covered by adsorbed or complexed enzyme (dimensionless), *i* is the fraction of total enzyme that is active (dimensionless), and $\varphi$ is the fraction of total cellulose sites which are free (dimensionless).

Unlike the classic Michaelis–Menten model, the HCH-1 model includes a parameter *ɛ* that describes the number of reactive sites covered by the enzymes [3, 7]. Furthermore, the HCH-1 model includes non-competitive inhibition by sugar products.

Unlike empirical models that apply only in the range where the data were taken, the HCH-1 model is mechanistic (Fig. 1) and, therefore, has broader applicability. As a mechanistic model, it applies to individual enzyme species; however, it has also been applied successfully to an enzyme cocktail in which the mixture is treated as a single “lumped” enzyme. Using the initial-rate data for pretreated biomass hydrolyzed by an enzyme cocktail, Brown et al. [7] compared mechanistic models and showed that the HCH-1 model provided the best fit to experimental data.

Previous studies show that, at high degrees of conversion, the hydrolysis rate drops by 2–3 orders of magnitude [8, 9]. The following factors contribute to the decreasing hydrolysis rates: (1) enzyme deactivation, (2) product inhibition, (3) decreased substrate reactivity, (4) decreased substrate accessibility, and (5) decreased synergism between cellulases [10]. In short-term enzymatic hydrolysis, these factors are not important and, therefore, are usually not incorporated into models that predict initial rates. However, in long-term batch saccharification, the reaction time is usually 3 to 5 days. As the reaction proceeds, the coefficients in short-term enzymatic hydrolysis models, such as HCH-1, may change because of the enumerated factors above. To describe long-term integrated enzymatic hydrolysis, the initial-rate models must be modified.

In this study, the original HCH-1 model was modified to describe 10-day enzymatic cellulose hydrolysis with commercial enzyme cocktail CTec2. The HCH-1 mechanism (Fig. 1) applies to individual enzymes in the cocktail; however, modeling each enzyme component is exceedingly complex. Understanding the kinetics of each enzyme component would be useful when optimizing the cocktail; however, this study uses a cocktail with defined components. Our approach is to treat the enzyme cocktail as a single “lumped” enzyme; hence, the resulting “lumped” parameters reflect the collective kinetics of the cocktail, not the individual components. To describe long-term enzymatic hydrolysis, this study investigates the relationships between the “lumped” parameters in the HCH-1 model and substrate conversion. The sensitivities of parameters in the modified model were analyzed. In addition, literature models for long-term (> 48 h) enzymatic hydrolysis were summarized and compared to the modified HCH-1 model.

## Materials and methods

### Materials

#### Substrate

The substrate used for all experiments was α-cellulose (Sigma-Aldrich, C8002). Compositional analysis showed that the substrate contained glucan 78.5% and xylan 14.4% [11].

#### Enzyme

The enzyme used in this study was Novozymes Cellic^®^ CTec2 (lot# VCPI 0007), a blend of aggressive cellulases with high levels of β-glucosidases and hemicellulases that degrade lignocellulose into sugars [12]. The protein concentration was determined to be 294 mg protein/mL with Pierce BCA assay [11]. Before use, the enzyme solution was diluted ten times with deionized (DI) water.

#### Citrate buffer

To maintain relatively high enzyme activity, citrate buffer (0.1 M) with a pH of 4.8 was used in enzymatic hydrolysis experiments. To prepare the buffer, citric acid monohydrate and trisodium citrate dihydrate were added to DI water.

#### Antibiotics

To prevent the growth of contaminating microorganisms that could consume produced sugars, an antibiotic cocktail was added. To prepare the solutions, tetracycline powder was dissolved in an aqueous solution of 70% ethanol at 10 g/L and cycloheximide powder was dissolved in DI water at 10 g/L.

### Enzymatic hydrolysis

In the enzymatic hydrolysis experiments, desired amounts of α-cellulose, glucose, and DI water together with 125 mL citrate buffer, 2 mL tetracycline solution, and 1.5 mL cycloheximide solution were added to a 1-L centrifuge bottle in sequence and then preheated. When the mixture reached 50 °C, enzymes were added. Then, the centrifuge bottle (total working volume of 250 mL) was placed in the incubator at 50 °C for 10 days with an axial rotation of 2 rpm. Liquid samples of 0.5 mL were taken periodically and submerged in boiling water for 20 min to deactivate the enzymes (note: the volume of liquid sample is small relative to the total slurry volume, so it is assumed to have a negligible impact on substrate concentration). Then, to determine the glucose concentration, the samples were filtered and analyzed by a high-performance liquid chromatography (HPLC), which was equipped with a pair of de-ashing guard columns (Bio-Rad Micro-Guard de-ashing cartridges, 30 mm × 4.6 mm) and an HPLC carbohydrate analysis column (Bio-Rad Aminex HPX-87P, 300 mm × 7.8 mm).

### Selection of hydrolysis conditions

#### Experiments for model fitness

Based on our previous study [11], 16 enzymatic hydrolysis conditions were tested including four different substrate concentrations (40, 60, 80, and 100 g/L), two different enzyme loadings (2 and 5 mg protein/g of dry biomass (mg/g)), and two different initial glucose concentrations (0 and 33 g/L).

#### Experiments for model predictions

Three enzymatic hydrolysis conditions—which were different from the conditions used for model fitness—were tested for model predictions: (1) substrate concentration: 40 g/L, enzyme loading: 1 mg/g, initial glucose concentration: 0 g/L; (2) substrate concentration: 70 g/L, enzyme loading: 1 mg/g, initial glucose concentration: 28 g/L; (3) substrate concentration: 100 g/L, enzyme loading: 5 mg/g, initial glucose concentration: 28 g/L.

### Enzyme stability

Enzyme stability was measured by quantifying the soluble protein concentration over the course of 20 days. In this experiment, the desired amount of CTec2 was added to the preheated mixture of citrate buffer, DI water, and antibiotic cocktail. The resulting solution was placed in the incubator at 50 °C. Samples of 0.5 mL were taken periodically and then centrifuged at 13,000 rpm for 10 min. The protein concentration of the supernatant was measured by the Pierce BCA method.

### Modification of HCH-1 model

#### Simulation of enzyme stability

Wallace et al. [13] reported that unproductive binding with lignin and thermal deactivation may play a significant role in enzyme deactivation. Considering the substrate used in this study is lignin-free, we assume that enzyme deactivation is solely due to thermal deactivation. Rosales-Calderon et al. [14] observed that the protein concentration of a mixture of glucanase and β-glucosidase dropped 34% after incubating at 50 °C for 4 days. It was hypothesized that the enzyme proteins suffered a structural change at 50 °C, which led to protein aggregation and precipitation. Additives, whose concentration was assumed constant and proportional to the initial enzyme protein concentration, were supposed to be present in the cocktail to stabilize the enzyme protein against aggregation. Equation 2 incorporates the presence of additives and is proposed to model protein stability [14]. The development of Eq. 2 is described in detail by Rosales-Calderon et al. [14].

2 $$\begin{array}{*{20}c} { - \frac{{\left. {{\text{d}}[E} \right]}}{{{\text{d}}t}} = k_{1} \left[ E \right] - k_{2} \left( {\left[ {E_{0} } \right] - \left[ E \right]} \right)\left[ {E_{0} } \right]} \\ \end{array} ,$$

where *E* is the native enzyme protein concentration (g/L), *E*_0_ is the initial enzyme protein concentration (g/L), and *k*_1_ and *k*_2_ are the rate constants (h^−1^).

The stability of CTec2 with three different initial concentrations was tested. Equation 2 was used to fit the experimental data.

#### Modification of HCH-1 model

**Fig. 2:**
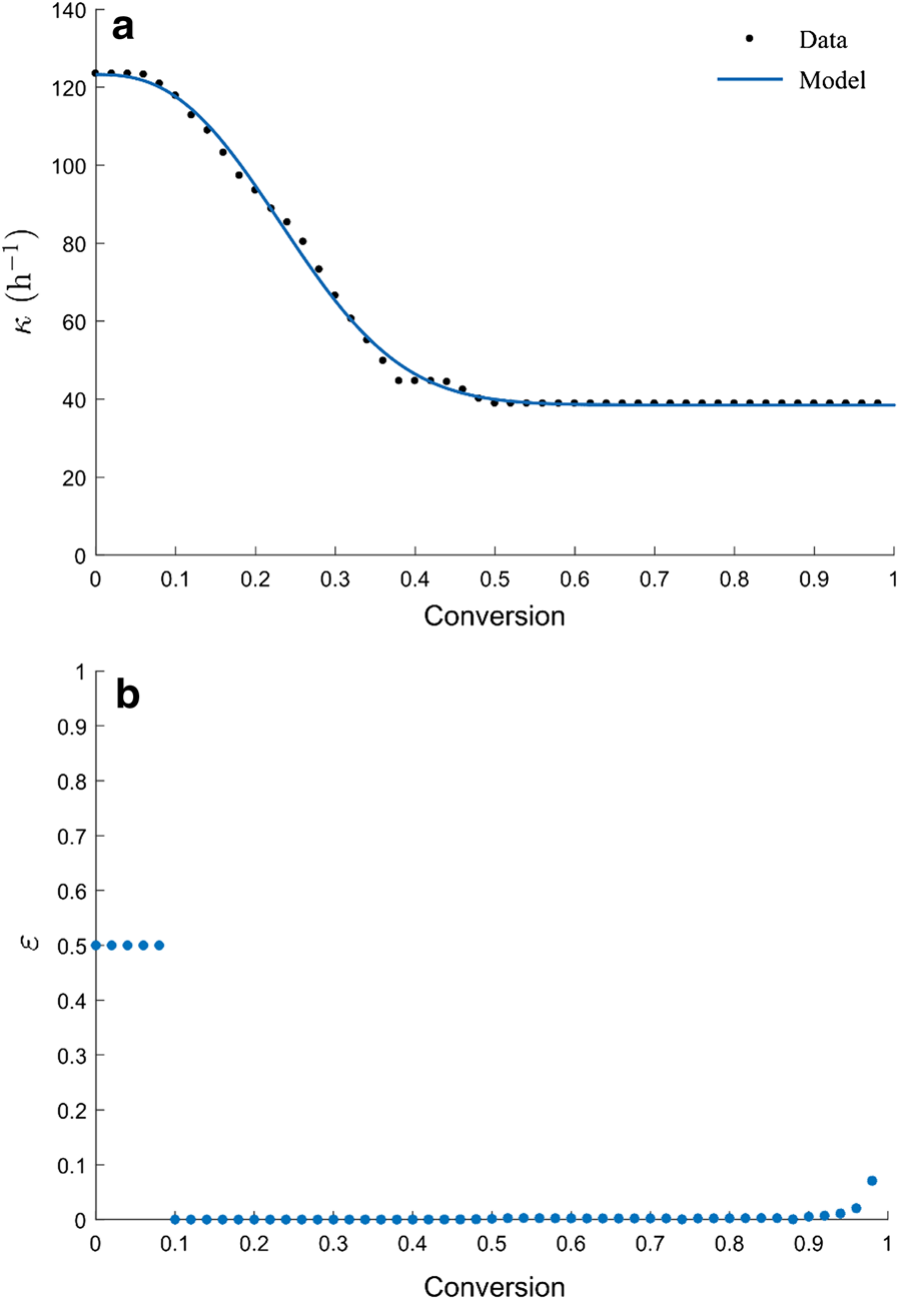
**a** The relationship between parameter *κ* and conversion *x.* **b** The relationship between parameter *ε* and conversion *x*

**Fig. 3:**
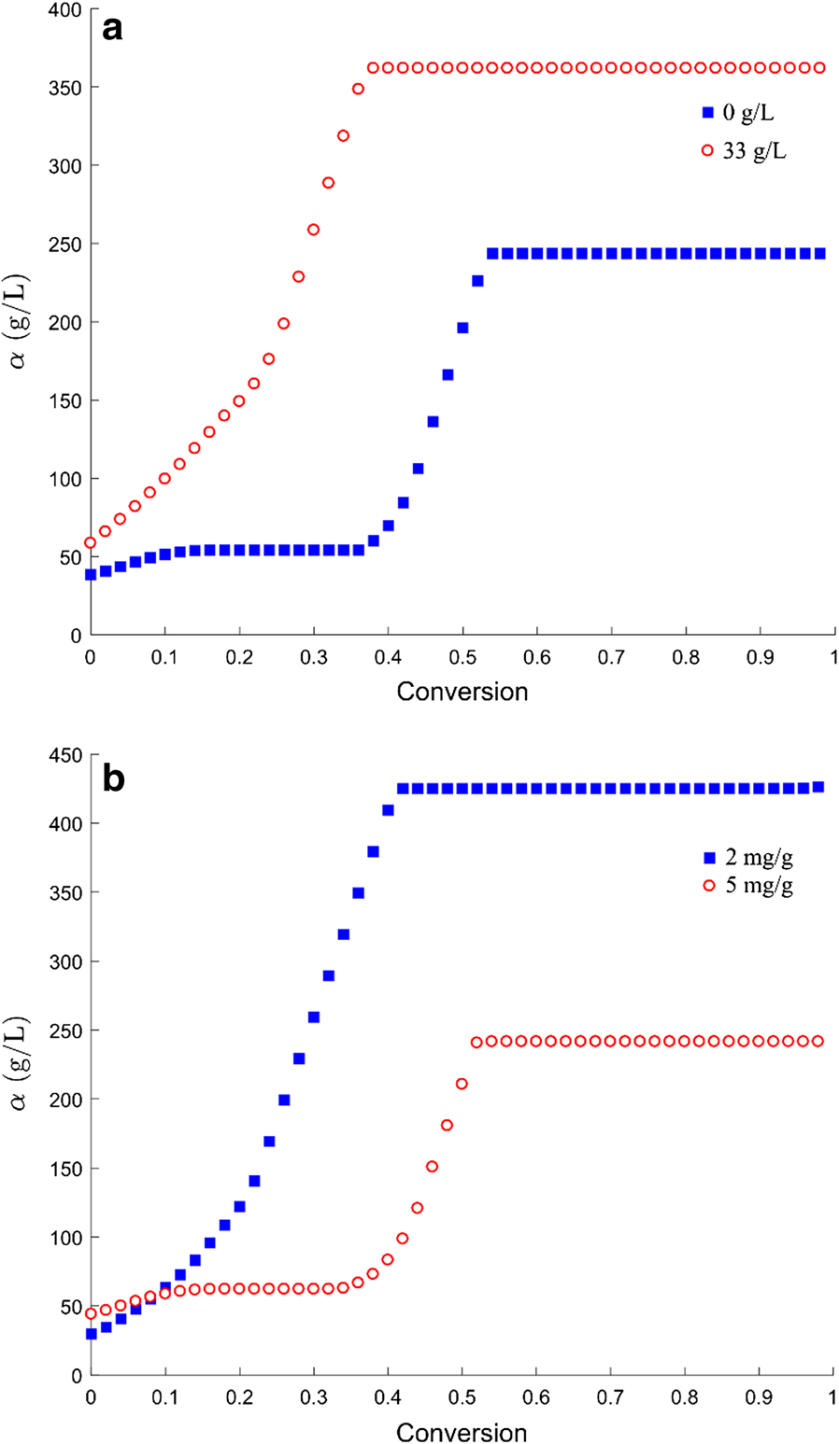
The relationship between parameter *α* and conversion *x* with **a** different initial glucose concentrations and **b** different enzyme loadings

The HCH-1 model was modified by the following steps:

Step 1: Use an empirical equation (Eq. 3) to fit the experimental data of the 16 enzymatic hydrolysis conditions (“Experiments for model fitness” section) with high accuracy. This smoothed version of the data provides the reaction rates needed to fit the parameters in the modified HCH-1 model of enzymatic hydrolysis.
  3 $$\begin{aligned} { \frac{{\left. {{\text{d}}[G_{1} } \right]}}{{{\text{d}}t}} = \frac{{3.7798\left( {\left[ {G_{x}^{0} } \right] - \left[ {G_{1} } \right]} \right)^{0.6410} \left( {\frac{{\left[ {E_{0} } \right]\left( {0.0574\left[ {E_{0} } \right] + 0.4370\exp \left( { - t\left( {0.4370 + 0.0574\left[ {E_{0} } \right]} \right)} \right)} \right)}}{{0.4370 + 0.0574\left[ {E_{0} } \right]}}} \right)^{0.8500} }}{{1 + 0.0247\left[ {G_{1} } \right]^{1.1579} }}} \\, \end{aligned} $$
  where $$\begin{array}{*{20}c} { G_{x}^{0} } \\ \end{array}$$ is the initial cellulose concentration (g/L, equivalent to glucose).
  Equation 3 was developed based on the integrated version of Eq. 2 and an empirical model for batch fermentation [15]. Detailed development of this equation is described in Additional file 1. To fit the parameters, Eq. 3 was solved with the *ode45* routine in MATLAB and nonlinear optimization was achieved by the *fmincon* routine. The objective function was the sum of square errors (SSE), which is the sum of the squared difference between experimental data and the predicted value [16]. The optimal set of parameters corresponds to the smallest SSE value. This empirical correlation of the data provided a coefficient of determination *R*^2^ = 0.994.
Step 2: Divide substrate conversion (from 0 to 1) into 50 equal segments. Using Eq. 3, calculate the reaction rate at each conversion and get a 16 × 50 data set.
Step 3: Determine product inhibition.
  The inhibition parameter *i* in the HCH-1 model was calculated by determining the initial velocities with and without inhibitor (Eq. 4) [17]. To estimate this value, the same enzyme and cellulose concentrations should be used.
  4 $$\begin{array}{*{20}c} { i = \frac{{V_{{{\text{with}}\;{\text{inhibitor}}}} }}{{V_{{{\text{no}}\;{\text{inhibitor}}}} }}} \\ \end{array} = \frac{{\left[ {\frac{{\kappa \left[ {G_{x}^{0} } \right]\left[ E \right]}}{{\alpha + \left[ {G_{x}^{0} } \right] + \varepsilon \left[ E \right]}}} \right]i}}{{\frac{{\kappa \left[ {G_{x}^{0} } \right]\left[ E \right]}}{{\alpha + \left[ {G_{x}^{0} } \right] + \varepsilon \left[ E \right]}}}}$$
  The inhibition of enzymatic hydrolysis by cellobiose was not considered in this study, because CTec2 contains a high level of β-glucosidase that rapidly converts produced cellobiose into glucose; the cellobiose peak was not found in the HPLC results.
  For a single inhibitor, the inhibition parameter *i* is expressed in Eq. 5 and the glucose binding constant *β*_1_ is calculated with Eq. 6.
  5 $$\begin{array}{*{20}c} { i = \frac{1}{{\left. {1 + \beta_{1} [G_{1} } \right]}}} \\ \end{array}$$
  6 $$\begin{array}{*{20}c} { \beta_{1} = \frac{{\left( {1 - i} \right)}}{{i\left[ {G_{1} } \right]}}} \\ \end{array}$$
Step 4: Use the HCH-1 model to fit the 16 reaction conditions at each conversion, and determine the best-fit coefficients *κ*, *α*, and *ɛ.*
Step 5: Determine the relationship between parameter *κ* and conversion *x.*
  Figure 2a presents the relationship between parameter κ in the HCH-1 model and substrate conversion *x*. The data were obtained from Steps 1–4. As shown in the figure, κ drops very fast in the beginning and then stabilizes after conversion reaches 0.38. Nidetzky and Steiner [18] assumed that cellulose consists of an easily hydrolysable part and a recalcitrant part. Based on their two-substrate hypothesis, the authors derived a mathematical model to describe the kinetics of cellulose hydrolysis. According to the simulation results, the obtained rate constant for easily hydrolysable cellulose was much higher than that of recalcitrant cellulose. Using α-cellulose as substrate, they determined that the fraction of easily hydrolysable cellulose was 0.3 [18]. Figure 2a can be explained by this hypothesis, but the rate constant for the easily hydrolysable cellulose decreases as conversion increases instead of being constant. In our experiments, the fraction of easily hydrolysable cellulose (0.38) is close to the result in [18].
  Equation 7 was developed to describe the relationship between parameter *κ* and conversion *x.*
  7 $$\begin{array}{*{20}c} { \kappa = \frac{{k_{3} }}{{\left( {1 + x^{{k_{4} }} } \right)^{{k_{5} }} }} + k_{6} } \\ \end{array} ,$$
  where *x* is the substrate conversion, *k*_3_, *k*_4_, *k*_5_, and *k*_6_ are the parameters.
  The conversion *x* in the denominator is used to describe the negative effect of conversion on the rate constant. The parameter $k_{6}$ is considered as the rate constant for recalcitrant cellulose. The parameter *k*_3_ is used to describe the difference in rate constants between the easily hydrolysable cellulose (initial) and recalcitrant cellulose (height of the curve). The parameters $k_{4} \;{\text{and}}\;k_{5}$ are used to describe the decrease rate of the rate constant (steepness of the curve) for the easily hydrolysable part. To fit the data, the MATLAB curve fitting tool was used and a coefficient of determination *R*^2^ = 0.998 was acquired. The values of parameters *k*_3_, *k*_4_, *k*_5_, and *k*_6_ were determined in this step.
Step 6: Determine the relationship between parameter *ε* and conversion *x.*
  Figure 2b shows the relationship between parameter *ε* in the HCH-1 model and conversion *x*. As shown in the figure, parameter *ε* has a very narrow range (0–0.5) over the entire conversion and remains almost unchanged (nearly zero) at conversion 0.1–0.95. Therefore, in this study, parameter *ε* is assumed not to be affected by conversion and its optimal value should be close to zero. Brown and Holtzapple [19] reported that if [$$\begin{array}{*{20}c} { G_{x}^{0} } \\ \end{array}$$]/[*E*_0_] > 35, assuming *ε *= 0 would not introduce considerable error (< 1%) (note: in our study, [$$\begin{array}{*{20}c} { G_{x}^{0} } \\ \end{array}$$]/[*E*_0_] ≥ 200). The parameter *ε* is needed only at high enzyme loadings. In industrial-scale saccharification, considering the high cost of enzymes, the enzyme dosage must be very low; therefore, if modeling a commercial process, the value of *ε* can be set as zero.
Step 7: Determine the relationship between parameter *α* and conversion *x*.
  The parameter *α* in the original HCH-1 model may be expressed by Eq. 8, which is related to enzyme adsorption:
  8 $$\begin{array}{*{20}c} {\alpha = \frac{{\left[ {E^{f} } \right]\left[ {G_{x}^{f} } \right]}}{{\left[ {E^{a} } \right] + \left[ {EG_{x} } \right]}}} \\ \end{array} .$$
  Kumar and Wyman [20] showed that glucose addition and enzyme dosage can affect the percentage of cellulase adsorption. Therefore, besides the impact of conversion, the effects of glucose and enzyme concentration on the value of *α* were tested. Using the best-fit coefficients *κ* and *ɛ* obtained from Step 4, two optimal *α* values corresponding to two initial glucose concentrations were determined by fitting the data (eight data at each initial glucose concentration) from Step 2 at each conversion with the HCH-1 model (Fig. 3a). Another two optimal *α* values corresponding to two enzyme concentrations were determined by repeating this procedure at each conversion (Fig. 3b). As shown in Fig. 3, as the reaction proceeds, the value of *α* increases and then is unchanged when the conversion reaches a certain point. It is obvious that high initial glucose concentration and low enzyme dosage improve the value of *α* significantly over the entire conversion range. Equation 9 was proposed to describe the relationship among *α,* conversion *x*, enzyme concentration *E*, and glucose concentration *G*_1_. As shown in Fig. 3, all four curves show an “S” shape; therefore, the sigmoid function—which has an S-shaped curve—was used. The core structure of Eq. 9 is a sigmoidal function that describes the relationship between parameter *α* and conversion *x* (note: *a*_2_ and *a*_3_ are the parameters of the sigmoid function). In addition, because of the significant effect of glucose and enzyme concentrations on the value of *α*, the terms [*G*_1_] and [*E*] were included in the numerator and denominator of the sigmoid function, respectively. The parameter *a*_1_ was added to describe the weight of terms [*G*_1_] and [*E*].
  9 $$\begin{array}{*{20}c} { \alpha = \frac{{a_{1} \left[ {G_{1} } \right]}}{{\left[ E \right]\left( {1 + { \exp }\left( { - a_{2} x + a_{3} } \right)} \right)}}} \\ \end{array} ,$$
  where *a*_1_, *a*_2_, and *a*_3_ are the parameters.
Step 8: Modify HCH-1 model.
  Summarizing the proposed equations, Eq. 10 is the modified HCH-1 model, where *k*_1_, *k*_2_, *k*_3_, *k*_4_, *k*_5_, *k*_6_, *a*_1_, *a*_2_, *a*_3_, *ε*, and *β*_1_ are parameters. Estimates for *k*_1_, *k*_2_, *k*_3_, *k*_4_, *k*_5_, *k*_6_, and *β*_1_ were determined in previous steps. In this step, the optimal values of *a*_1_, *a*_2_, *a*_3_, and *ε* were determined by simultaneously fitting the experimental data of the 16 enzymatic hydrolysis conditions (Section: Experiments for model fitness) with Eq. 10.
  $$\frac{{\left. {d[G_{1} } \right]}}{dt} = \frac{{\kappa \left[ {G_{x} } \right]\left[ E \right]i}}{{\alpha + \varphi \left[ {G_{x} } \right] + \varepsilon \left[ E \right]}},$$
  where,
  $$i = \frac{1}{{\left. {1 + \beta_{1} [G_{1} } \right]}}$$
  $$\begin{array}{*{20}c} {\varphi = \frac{{\left[ {G_{x} } \right] - \alpha - \varepsilon \left[ E \right] + \sqrt {\left( {\left[ {G_{x} } \right] - \alpha - \varepsilon \left[ E \right]} \right)^{2} + 4\alpha \left[ {G_{x} } \right]} }}{{2\left[ {G_{x} } \right]}}} \\ \end{array}$$
  $$- \frac{{\left. {d[E} \right]}}{dt} = k_{1} \left[ E \right] - k_{2} \left( {\left[ {E_{0} } \right] - \left[ E \right]} \right)\left[ {E_{0} } \right]$$
  $$\kappa = \frac{{k_{3} }}{{\left( {1 + x^{{k_{4} }} } \right)^{{k_{5} }} }} + k_{6}$$
  10 $$\begin{array}{*{20}c} { \alpha = \frac{{a_{1} \left[ {G_{1} } \right]}}{{\left[ E \right]\left( {1 + { \exp }\left( { - a_{2} x + a_{3} } \right)} \right)}}} \\ \end{array} .$$
  Integration of the differential equations described in Eq. 10 was performed using the same numerical methods described in Step 1.

### Sensitivity analysis

#### Local sensitivity analysis

Local sensitivity analysis assesses the local impact of variation in input factors on model outputs. To do this analysis, the direct differential method [21] was used by calculating the sensitivity indices (Eq. 11). The sensitivities of parameters *k*_1_, *k*_2_, and *β*_1_ were not analyzed, because their values were obtained from independent experiment or calculation.

11 $$\begin{array}{*{20}c} { S_{{p_{j} }} = \frac{\partial y}{{\partial p_{j} }}\frac{{p_{j} }}{y}} \\ \end{array},$$

where $S_{{p_{j} }}$ is the non-dimensional sensitivity index of the *j*th parameter, *y* is the glucose concentration (g/L), and *p*_*j*_ is the *j*th parameter in the parameter vector *p.*

#### Global sensitivity analysis

Local sensitivity only analyzes the sensitivity of a solution close to the optimal parameter set. In contrast, global sensitivity analysis assesses the sensitivity of the model for the full range of possible parameter values [16]. In addition, global sensitivity indices can evaluate the combined impact of multiple parameters on model output.

To calculate a global sensitivity index, a normally distributed search of parameter space using the Monte Carlo method was performed and subsequent analysis of the variance in the model outputs was used. In this study, two global sensitivity indices were calculated: first-order index and total-effect index [16, 22]. The first-order index measures the effect of the parameter of interest alone on the output variance. The total-effect index accounts for not only the effect of the parameter of interest, but also interactions between the other parameters and the parameter of interest at any order.

### Model evaluation

The modified HCH-1 model was compared with the literature models for long-term enzymatic hydrolysis. As described in Step 1, the same differential equation integration method, nonlinear optimization constraint algorithm, and objective function were used. The Akaike information criterion (AIC) was used to evaluate model quality for the experimental data. The corrected version of AIC (Eq. 12) was used, because the number of observations was not large enough:

12 $$\begin{array}{*{20}c} {{\text{AIC}}_{C} = N \cdot \ln \left( {\frac{\text{SSE}}{N}} \right) + 2\left( {P + 1} \right) + 2\frac{{\left. {\left( {P + 1} \right)(P + 2} \right)}}{N - P}} \\ \end{array} ,$$

where *N* is the number of observations, *P* is the number of model parameters, and SSE is the sum square error.

## Results and discussion

### Enzyme deactivation

**Fig. 4:**
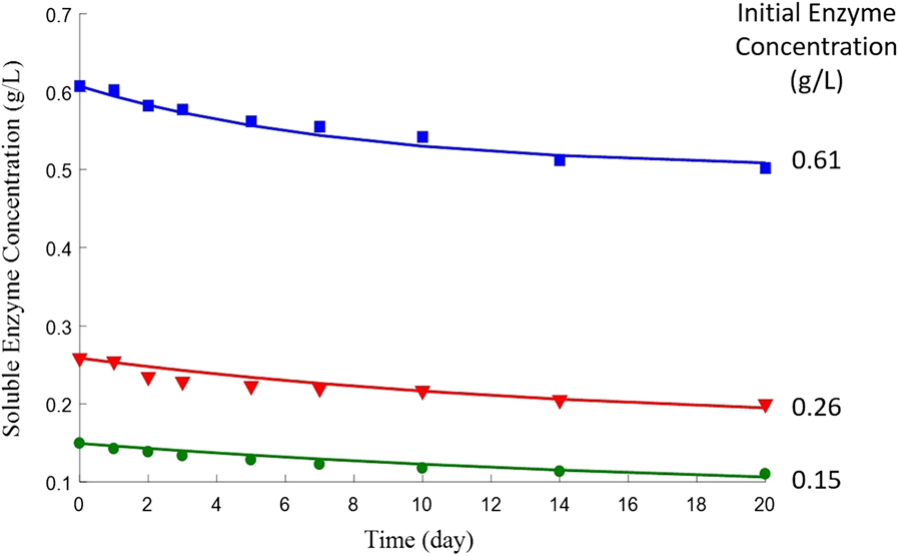
Time profiles and model predictions for soluble CTec2 protein concentration at 50 °C. Experimental data are presented by the markers and the optimal fit by the solid lines

Figure 4 shows that, after incubating at 50 °C for 20 days, soluble protein concentrations of CTec2 dropped to 74, 77, and 83% of their initial values of 0.15, 0.26, and 0.61 g/L, respectively. This result is consistent with a previous study [14] that shows higher initial protein concentrations favor lower deactivation rates and supports the additive hypothesis. Equation 2 successfully describes the time profiles of CTec2 protein concentration with a coefficient of determination *R*^2^ = 0.999. The rate constants in Eq. 2 were determined to be *k*_1_ = 0.0225 h^−1^ and *k*_2_ = 0.1740 L/(g h). It should be noted that the modified HCH-1 model is a “lumped” model, the performance of each enzyme was not modeled individually; therefore, the stability of each component in the enzyme cocktail was not investigated. Equation 2 describes the overall deactivation of the cocktail.

### Production inhibition

Table 1 lists the values of glucose-binding constant calculated from various reaction conditions. The eight *β*_1_ values are very close to each other and have a standard deviation of 6 × 10^−6^ L/g. The mean value 0.0429 L/g is considered to be the “true” *β*_1_ value and is used for later calculations.

**Table 1:** Glucose-binding constant from various reaction conditions

Reaction condition		*β*_1_ (L/g)
Substrate concentration (g/L)	Enzyme loading (mg/g)	
40	2	0.042908
40	5	0.042915
60	2	0.042912
60	5	0.042918
80	2	0.042920
80	5	0.042923
100	2	0.042922
100	5	0.042925

### Model validation

**Fig. 5:**
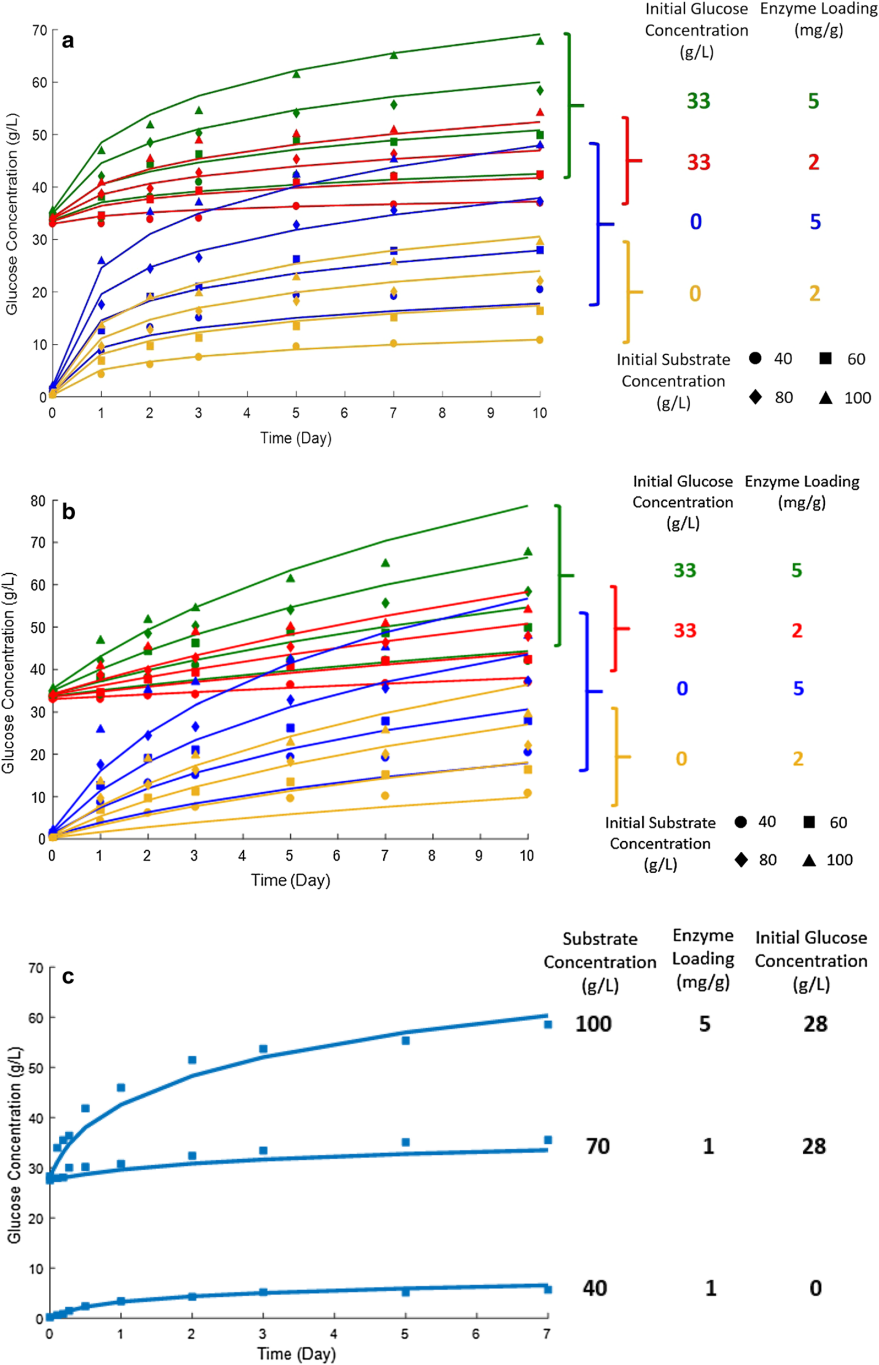
**a** Time profiles and modified HCH-1 model fitting results for enzymatic hydrolysis of α-cellulose. **b** Time profiles and original HCH-1 model fitting results for enzymatic hydrolysis of α-cellulose. **c** Time profiles and modified HCH-1 model predictions for enzymatic hydrolysis of α-cellulose. Experimental data are presented by the markers and the values of parameters are from Table 2

Figure 5a shows the experimental data and modified HCH-1 model fitting results for enzymatic hydrolysis with 16 reaction conditions (“Experiments for model fitness” section). Table 2 shows the values of the parameters obtained from the previous section. The model simulation provided the coefficient of determination *R*^2^ = 0.992, which indicates that the modified HCH-1 model describes enzymatic hydrolysis of α-cellulose very well.

**Table 2:** Optimal parameter estimates for the modified HCH-1 model

Parameter	Value	Unit
*k*_1_	0.0225	h^−1^
*k*_2_	0.1740	L/(g h)
*k*_3_	84.7500	h^−1^
*k*_4_	2.5800	Dimensionless
*k*_5_	26.3600	Dimensionless
*k*_6_	38.5000	h^−1^
*a*_1_	1.6791	g/L
*a*_2_	31.1485	Dimensionless
*a*_3_	2.8452	Dimensionless
*ε*	5.5248 × 10^−5^	Dimensionless
*β*_1_	0.0429	L/g

As a comparison, Fig. 5b shows the original HCH-1 model fit to the experimental data with 16 reaction conditions (“Experiments for model fitness” section). The value of *β*_1_ (0.0429 L/g) was obtained from the previous section (product inhibition). The optimal values (*α* = 2.0776 × 10^6^ g/L, *κ* = 9.2889 × 10^5^ h^−1^, and *ɛ* = 0.9996) were determined (note: because the original HCH-1 model was not developed for integrated cellulose hydrolysis, these parameter values are not be meaningful). The model simulation provided the coefficient of determination *R*^2^ = 0.947. The calculated SSE and AICc are listed in Table 3.

**Table 3:** Comparison of long-term enzymatic hydrolysis models

Model	*N* (Obs)	Parameter	SSE	AICc	Methodology
Modified HCH-1 (16)	112	11	236.7	110.9	Ads
Holtzapple et al. [3] (original HCH-1)	112	4	1630.7	310.5	Ads
Drissen et al. [26]	112	11	600.8	215.2	Ads, M–M
Fan and Lee [27]	112	11	679.7	229.0	Ads
Liao et al. [28]	112	5	1313.7	288.5	Ads
Peri et al. [21]	112	12	2657.6	384.3	Ads, M–M
Fenila and Shastri [5]	112	22	2080.9	385.5	Ads, M–M
Kadam et al. [4]	112	18	2338.6	386.4	Ads, M–M
Gusakov et al. [29]	112	16	2879.2	404.0	M–M
Philippidis et al. [30]	112	7	9139.8	510.4	Ads, M–M
Modified HCH-1 (8)^a^	56	11	115.2	71.3	Ads
Shen and Agblevor [31]^a^	56	4	493.8	133.1	Ads
Zhang et al. [32]^a^	56	3	692.6	149.6	Ads
Rosales-Calderon et al. [33]^a^	56	3	692.6	149.6	Ads
Nidetzky and Steiner [18]^a^	56	5	743.9	158.5	Ads

*M–M* Michaelis–Menten kinetics, *Ads* adsorption-based approach

^a^Only eight reaction conditions were fit [0 g/L initial glucose, four substrate concentrations (40, 60, 80, and 100 g/L) × two enzyme loadings (2 and 5 mg/g)]

### Model predictions

The modified HCH-1 model (Eq. 10) was used to predict the experimental results of the three conditions described in “Experiments for model prediction.” The parameter values were obtained from the fitness of the 16 conditions (Table 2). The experimental and predicted results are shown in Fig. 5c. The simulation provided the coefficient of determination *R*^2^ = 0.991, which indicates that the modified HCH-1 model predicts enzymatic hydrolysis of α-cellulose with high accuracy.

### Sensitivity analysis

**Fig. 6:**
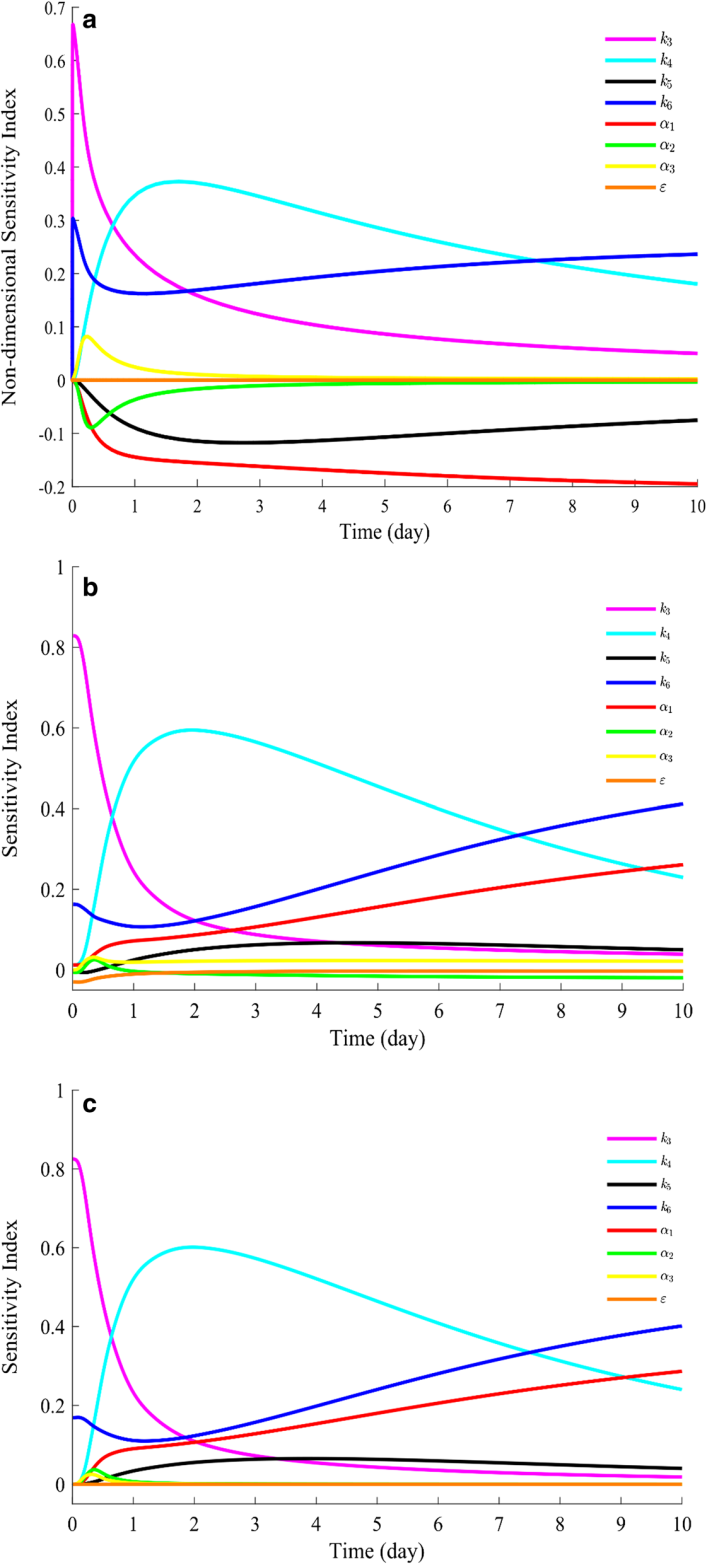
**a** Local sensitivity analysis of the modified HCH-1 model at the optimal solution. Global sensitivity analysis of the modified HCH-1 model over the course of 10 days, **b** first-order indices; **c** total-effect indices

To explore the controlling factors in the proposed model at different hydrolysis stages, local and global sensitivity analyses were performed. Figure 6a shows the parameter sensitivity indices from local sensitivity analysis of the modified HCH-1 model over the course of 10 days. As shown in the figure, the sensitivity of *k*_3_ drops to nearly 10% of its initial value at day 10. The sensitivity of *k*_4_ increases first and reaches up to 0.4 at around day 1, and then slightly decreases from day 2 to day 10. For the parameters about *α*, the sensitivity of *a*_1_ (absolute value) increases as the hydrolysis time increases. The sensitivities of *a*_2_ and *a*_3_ only change within the first several reaction days, and then are close to zero after day 3. The sensitivity of *ε* is close to zero during the entire reaction time.

Figure 6b, c shows the global sensitivity analysis results of the modified HCH-1 model. According to the figures, the first-order indices and total-effect indices of all variables are almost identical at any time, which means that the variance in this model is not related to any interaction between parameters. At the initial stage of hydrolysis, the variance in the model output only depends on *k*_3_ and *k*_6_. Then, the sensitivity index of *k*_3_ decreases very fast during the first 2 days, whereas *k*_4_ increases up to 0.6. From day 2 to day 10, the effects of *k*_6_ and *a*_1_ on the model increase. The variables *a*_2_, *a*_3_, and *ε* do not show significant effects on the variance in model predictions.

**Fig. 7:**
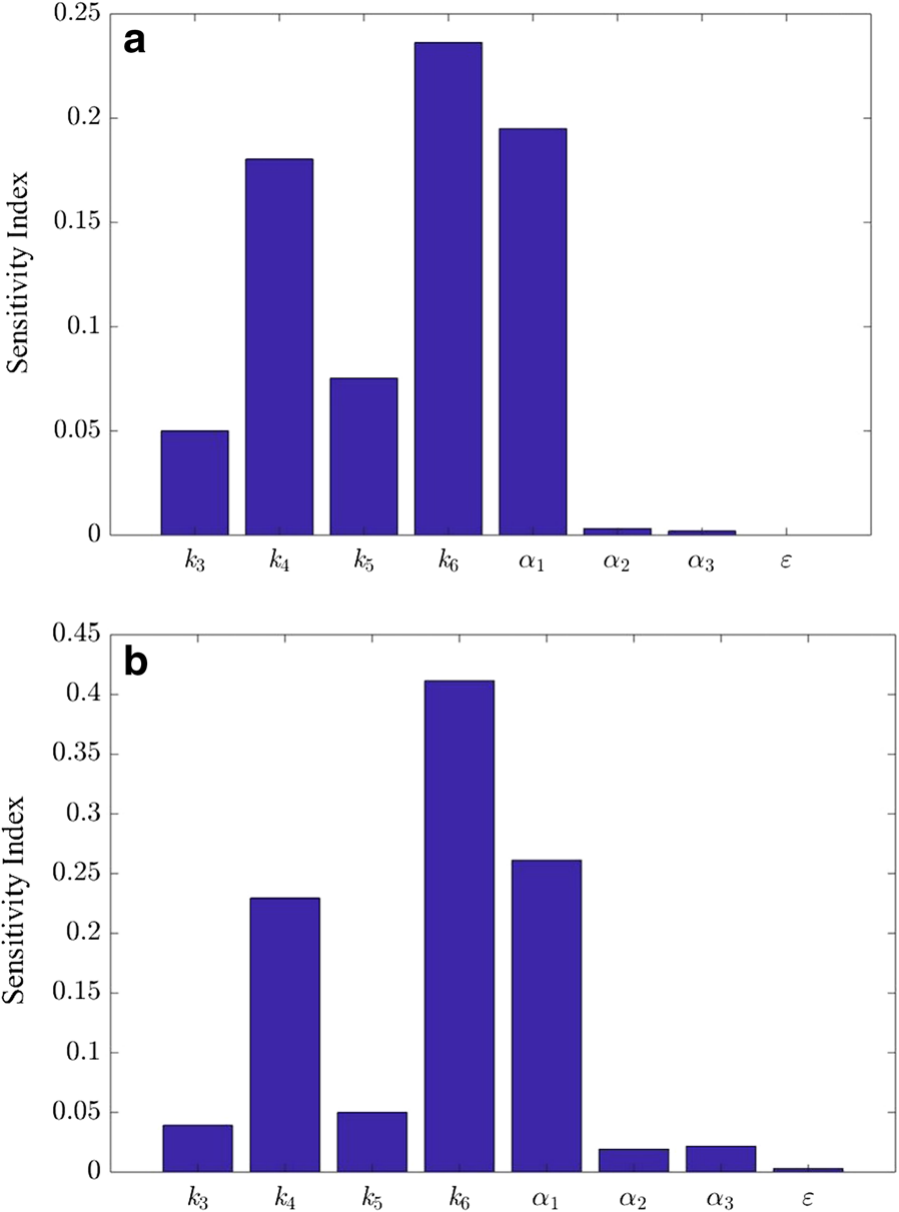
The local and global sensitivity indices of the modified HCH-1 model at day 10. **a** Local sensitivity analysis and **b** global sensitivity analysis (first-order indices)

According to Fig. 6, the local and global sensitivity analyses of the modified HCH-1 model show a similar trend during the entire reaction time. Figure 7 shows the sensitivity indices calculated from both analyses at day 10. The rankings of the eight sensitivity indices from both analyses are almost the same (*k*_6_ > *a*_1_ > *k*_4_ > *k*_5_ > *k*_3_ > *a*_2_ ≈ *a*_3_ ≈ *ε*).

The sensitivity analyses not only determine which parameters have the most influence on model results, but also verify the assumption in Step 6 that the parameter *ε* is not needed at low enzyme loadings. These analyses provide direction for further modification of the HCH-1 model to apply it to real-world lignocellulose that contains lignin.

### Model comparison

Based on the methodology used, the published mechanistic and semi-mechanistic models for cellulose and lignocellulose can be broadly divided into two classes: Michaelis–Menten and enzyme-adsorption models [10]. The models following Michaelis–Menten kinetics can also be divided into two subclasses: full Michaelis–Menten models (all rate equations follow Michaelis–Menten kinetics, including the steps of cellulose to cellobiose, cellulose to glucose, and cellobiose to glucose) and partial Michaelis–Menten models (only the step of cellobiose to glucose follows these kinetics). Models employing enzyme adsorption typically use Langmuir adsorption isotherms or the help of kinetic equations [10]. Some literature models incorporate both enzyme adsorption and Michaelis–Menten kinetics.

In this study, the published models for long-term enzymatic hydrolysis of cellulose and lignocellulose were fit to the experimental data using the numerical methods described in Step 1. Some models do not consider product inhibition. To make a fair comparison, these models were only fit to experimental conditions with no initial sugar added (0 g/L initial glucose; four substrate concentrations × two enzyme loadings). Some models teased out fine details in the elementary reaction steps and included some variables that were not determined in this study, such as exocellulase concentration and associated enzyme concentration [23–25]. These models are not included in this section. Table 3 summarizes the number of observations and parameters, calculated SSE and AICc values, and the methodology used for the published models. According to the table, the modified HCH-1 model has the least SSE and AICc values, which indicates that this model provides the best fit for long-term enzymatic hydrolysis of α-cellulose.

## Conclusion

The original HCH-1 model was modified to extend its application to integrated enzymatic hydrolysis; it performed well when fitting 10-day cellulose hydrolysis at various experimental conditions. Local and global sensitivity analyses were performed to determine the controlling parameters at different hydrolysis stages. Mechanistic (and semi-mechanistic) literature models for long-term enzymatic hydrolysis were compared with the modified HCH-1 model and evaluated by AICc. Comparison results show that the modified HCH-1 model provides the best description of enzymatic cellulose hydrolysis. The “lumped” modified HCH-1 model developed in this study has a simpler form and fewer parameters than mechanistic models of each enzyme component. When each enzyme is modeled separately, the kinetics is extremely complex with the potential to over-parameterize. For the specific commercial enzyme cocktail used in this study, excellent fits to the data were obtained without the need to model each enzyme component individually.

## Additional file

**Additional file 1.** Development of Eq. 3.

## Abbreviations

mg/g: mg protein/g of dry biomass
AIC: Akaike information criterion
SSE: sum of square errors
DI water: deionized water
M–M: Michaelis–Menten kinetics
Ads: adsorption-based approach
