# Supplementary material for: Development of modified HCH-1 kinetic model for long-term enzymatic cellulose hydrolysis and comparison with literature models
Source: Biotechnol Biofuels. 2019 Feb 18;12:34. doi: 10.1186/s13068-019-1371-5 (PMC6378734; doi:10.1186/s13068-019-1371-5)
Supplement: Supplementary file 1 — Additional file 1. Development of Eq. 3. [file 13068_2019_1371_MOESM1_ESM.doc]

**Additional Information: Development of Eq. 3**

The core structure of Eq. 3 was inspired by the empirical equation for batch fermentation (Eq. 13, [1]), where sugarcane bagasse and chicken manure were anaerobically fermented to carboxylic acids.


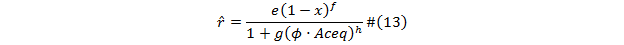


where,

*x* is the fraction conversion of volatile solid
*e*, *f*, *g*, and *h* are the empirical constants

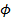
 is the ratio of total grams of carboxylic acid to total grams of acetic acid equivalents (*Aceq*).

This equation relates the specific reaction rate
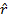
(*x*, *Aceq*) with *Aceq* concentration and conversion (*x*). The term (1 − *x*) is described as the “conversion penalty function,” which means the reaction rate decreases as the substrate is converted [1, 2]. The denominator term describes the inhibitory effect of product (*Aceq*) on reaction rate.

To model enzymatic hydrolysis process, Eq. 13 was modified to Eq. 14 with the following two major adjustments: (1) the effect of enzyme concentration
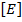
, and (2) the effect of substrate concentration
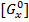
.


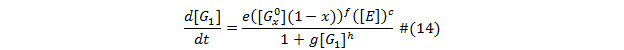


Solving Eq. 2 taking
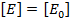
 (g/L), at
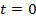
 yields:


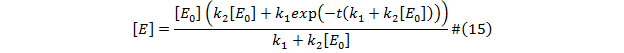


Eq. 16 can be obtained by replacing the term [*E*] in Eq. 14 with Eq. 15.


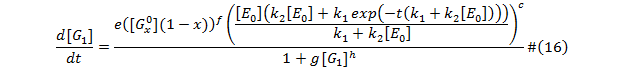


The optimal values of the parameters *e*, *f*, *g*, *h*, *c*,
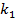
, and
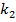
 were determined by fitting the experimental data of the 16 enzymatic hydrolysis conditions (Section: Experiments for model fitness) with Eq. 16 simultaneously. Eq. 3 was obtained by replacing the parameters in Eq. 16 with the optimal values.

**Reference**

1. Fu Z, Holtzapple MT. Anaerobic mixed-culture fermentation of aqueous ammonia-treated sugarcane bagasse in consolidated bioprocessing. Biotechnol Bioeng. 2010;106:216–27.

2. South CR, Lynd LR. Analysis of conversion of particulate biomass to ethanol in continuous solids retaining and cascade bioreactors. Appl Biochem Biotechnol. 1994;45:467.
